# Serial measurement of mood via text messaging in young people

**DOI:** 10.1186/s13034-020-0313-0

**Published:** 2020-01-29

**Authors:** Philip Hazell, Ben Balzer, Patrick Kelly, Karen Paxton, Catherine Hawke, Rebecca Ivers, Rachel Skinner, Georgina Luscombe, Katharine S. Steinbeck

**Affiliations:** 1 University of Sydney School of Medicine, Sydney, Australia; 2 University of New South Wales School of Public Health and Community Medicine, Sydney, Australia

**Keywords:** Adolescent, Depression, Surveys and questionnaires, Text messaging

## Abstract

**Background:**

To support longitudinal research into mood in adolescents we sought to assess the feasibility of collecting mood data via Short Message Service (SMS) over 3 years, and to investigate the relationship between SMS data and self-report measures of depression.

**Methods:**

Prospective cohort study of young people aged 9 to 14 years at baseline. Participants completed Short Mood and Feelings Questionnaire (SMFQ) and the Youth Self Report Anxious/Depressed ((YSR)/AD) and Withdrawn/Depressed (YSR/WD) scales at baseline and annually for 3 years. In addition, at 3 monthly intervals they responded to an SMS asking them to rate their mood from 0 to 9 (9 highest).

**Results:**

277 young people (43% female) completed all four waves of the survey. There was a 87% response rate to requests for SMS Mood ratings. Mean SMS Mood decreased over time for females (p = 0.006) but not males (p = 0.45). We found an inverse association between SMS Mood and the SMFQ, YSR/AD and YSR/WD, scales in females and the SMFQ and YSR/WD scales in males. 45% of participants reported at least one SMS Mood rating score below 5, while 5% reported clusters of low SMS scores. Clusters of low SMS Mood scores were associated with SMFQ scores in the clinical range at 24 (OR = 4.45) and 36 months (OR = 4.72), and YSR/WD in the clinical range at 36 months (OR = 4.61).

**Conclusions:**

SMS Mood ratings represent a feasible means to augment but not replace assessment of mood obtained using standard instruments.

## Background

The 12-month prevalence of Major Depressive Disorder among adolescents aged 13–18 years in the US is 7.5% [1]. The prevalence of depression increases with age through the adolescent period, more so in females than males [2]. Historically, the tracking of mood disorder over time has required the repeat administration of clinical interviews or mood questionnaires. The logistics of doing this limit the frequency with which assessments can be undertaken, risking the possibility that brief episodes will be missed. There is also limited capacity to determine time of onset and offset of disorder. Brief scales delivered via text messaging offer a promising alternative for capturing mood data. Text messaging via Short Message Service (SMS) has been demonstrated to be an ‘adolescent-friendly’ and effective method of maintaining young research participants in longitudinal studies as well as a useful reminder tool [3]. Due to high cellular phone ownership and the ubiquity of text messaging as a means of communication among adolescents, SMS may also be effective as a data collection tool in longitudinal research, provided that the information requested does not require a complex response. Simple and frequently obtained measures of mood in adolescents, if valid, afford the opportunity for a more fine-grained examination of the relationship between mood and other events such as puberty onset. In addition, such measures may provide a more accurate indication of the onset and duration of mood disorder in young people participating in longitudinal research. Text messaging offers a number of advantages over pen and paper methods of mood monitoring, as demonstrated in a study of high school students in Ireland [4]. Compliance rates are higher with SMS than pencil and paper, and the ratings are more likely to be contemporaneous rather than retrospective. Compliance is probably higher because SMS texting affords more privacy than pencil and paper methods, and because young people typically have a mobile phone with them, while the pencil and paper format requires carrying ‘special equipment’ [4]. SMS Mood monitoring has been validated against the Patient Health Questionnaire-9 (PHQ-9), a standardized measure of depression, in adults [5, 6], but not to our knowledge against depression measures designed for young people. Thirty-three adults received daily automated text messages measuring their mood (What is your mood right now on a scale of 1–9?) and inquiring about thoughts and activities as part of their participation in group cognitive behavioural therapy for depression in a public sector clinic. During this time, they also received a PHQ-9 each week that they attended the therapy group. There was a significant relationship between daily mood scores and 1-week average mood scores and PHQ-9 scores controlling for linear change in depression scores, but the relationship between the 2-week average mood and PHQ-9 scores was non-significant [6]. In a randomised controlled trial of acupuncture for depression in adults SMS Mood scores were obtained weekly for 16 weeks. At week 16 post-randomisation, 220 participants responded to the weekly depression text within 6 days of completing the PHQ-9 paper questionnaire. The two measures were moderately correlated at that point (Kendall’s tau-b = 57, p < 001) [7]. To our knowledge SMS has not been used to track mood over long periods in young people or adults.

The aim of the current study was to assess the feasibility of collecting mood data at intervals over a period of 3 years via text message, as well as to describe the patterns and stability of the data in a cohort of rural young people. A further aim was to investigate the relationship of the mood data to self-report measures of depression obtained at baseline and at yearly intervals for 3 years. We hypothesized that better SMS Mood ratings would be associated with fewer depression symptoms on standard rating scales. Finally, we sought to determine, through exploratory analyses, whether clusters or runs of low SMS Mood ratings are associated with subsequent scores in the ‘clinical’ range on other depression measures.

## Methods

The Adolescent Rural Cohort study of Hormones, health, Education, environments and Relationships (ARCHER) commenced in 2011, with the aim of examining the effect of pubertal hormone changes on adolescent health and well-being in a rural Australian cohort. Its protocol has been detailed previously [8]. Adolescents (9–14 years of age) and their parents were recruited from the community in two large regional towns in Australia, and their surrounding areas. Lack of parental competence in English or adolescent intellectual disability were exclusion criteria [8]. As part of the protocol, study participants completed self-report Short Mood and Feelings Questionnaires (SMFQ) [9] and youth self report (YSR) [10] questionnaires at baseline and at 12-monthly intervals for 3 years. In addition a text message was sent every 3 months via an automated SMS service to the nominated mobile phone asking the participant to rate their current mood.

The SMFQ is a 13-item subscale from a longer 33-item questionnaire (the original MFQ) validated for children and young people aged between 6 and 17 years. The questions asked on the survey are based on the Diagnostic and Statistical Manual criteria for depression. Respondents are asked to indicate the extent to which each item applied to them over the previous 2 weeks on the following scale: 0 (*not true*), 1 (*sometimes*), 2 (*true*). We defined caseness on the SMFQ as a score of 11 or greater as recommended by Patton et al. [11]. The YSR is a well validated and reliable assessment tool that measures emotional and mental wellbeing in 11–18 year olds. The scale contains 113 items, and respondents rated the extent to which each item applied to them on the following scale: 0 (*not true*), 1 (*somewhat or sometimes true*), 2 (*very true or often true*). The current analysis used scores from the Anxious/Depressed (YSR/AD) and Withdrawn/Depressed (YSR/WD) subscales. YSR subscale percentile scores were categorized to three levels as per the developers of the instrument: 0 (non-clinical), 1 (borderline), and 2 (clinical). To enable binary analyses the borderline and clinical categories were collapsed into one ‘clinical’ category. The SMS comprised a standard text: “Hi «First Name» «Last Name». On a scale from 0 to 9 (with 9 = best ever), what is your mood now? Thanks «Investigator Name».” This message is a linear analogue self-assessment scale of mood to assess mood fluctuation, modelled on previous SMS studies [5, 6]. Replies were sent to the automated service and downloaded to the study database.

## Statistical analyses

Comparisons between those adolescents who did or did not complete all four study waves were made using a Chi square analysis for gender and Mann–Whitney U tests (MW U z) for age and Tanner stage (due to significant distributional skew and ordinal data, respectively). Analyses over time of the rate of SMS Mood measure completion and the proportion ‘low’ range SMS Mood scores [see below for method] were explored using Cochran’s Q tests. Because the data comprised repeated measures we examined time effects on the four respective mood measure variables (SMS Mood, SMFQ, YSR/AD, YSR/WD) using linear regression models fitted via general estimating equations (GEEs) assuming an exchangeable correlation structure. For the SMS Mood scores the time effect analyses included 13 time points, and for SMFQ, YSR/AD and YSR/WD only four time points. Given known gender effects, analyses were repeated and results reported separately for males and females. A series of generalised linear models (GLMs) was conducted to explore the relationship between SMS Moods scores (measured at the four time points corresponding to the four study waves) and the other mood measures. Time and gender were included as covariates in these models, and analyses were repeated within each gender separately.

To determine whether clusters or runs of low SMS Mood ratings are associated with subsequent scores in the ‘clinical’ range on other depression measures we first needed to identify a suitable cut-point for the SMS Mood rating. Using the first 12 months of data Receiver Operating Curve analysis determined that a cut-score of 4 on SMS Mood ratings offered the best sensitivity and specificity for detecting clinical caseness on the SMFQ. Hence, a binary variable was created for SMS Mood, where a coding of 0 represented a mood rating of 5 or higher, and a coding of 1 represented low mood (≤ 4). For the purpose of analyses, we defined a cluster as three or more scores below 5 on the SMS Mood rating, separated by no more than one score either in the normal range or missing. The association between SMS Mood clusters and SMFQ scores in the clinical range was determined for each study wave by producing odds ratios with 95% confidence intervals. Data were analysed using SPSS Version 24. Alpha was set at 0.05 for all analyses.

## Results

### Participants

Baseline characteristics of the sample, and a comparison of those who did and did not complete all four waves of data collection, are summarized in Table 1. The median age of the 342 young participants at baseline was 11 years, 45% were female and 11% were Indigenous. The sample was representative of the region in terms of gender (proportion in region 49% female), but underrepresented youth of Indigenous background (proportion in region 17%). In comparison to the region, participating families were generally more likely to have at least one adult in employment (87% vs region 81%) and to own their own dwelling (80% vs region 68%). Parents were also more likely to have completed tertiary education (49% vs region parents 20%). Fifty-six percent of females and 49% of males rated themselves as pre-pubertal or early-pubertal (Tanner stage 1 to 2). Data were analysed for participants with responses to all four waves of 12 monthly data collection (n = 277, 119 (43%) female). Baseline differences between the group that completed the study and the non-completers in age, gender and self-reported Tanner stage were not statistically significant (see Table 1).

**Table 1:** Baseline characteristics of the sample

	Completers (n = 277)	Non-completers (n = 65)	Test statistic	p value
Female, n (%)	119 (43.0)	34 (52.3)	Χ^2^ = 1.86, df = 1	0.17
Age years, median (IQR)	11.6 (1.6)	11.9 (1.6)	MW U z = − 1.91	0.06
Tanner stage, median (IQR)				
Whole sample	2 (1)	3 (2)	MW U z = 0.80	0.43
Females	2 (1)	2 (2)	MW U z = 0.88	0.38
Males	3 (1)	3 (2)	MW U z = 0.46	0.65

### Descriptive data

**Fig. 1:**
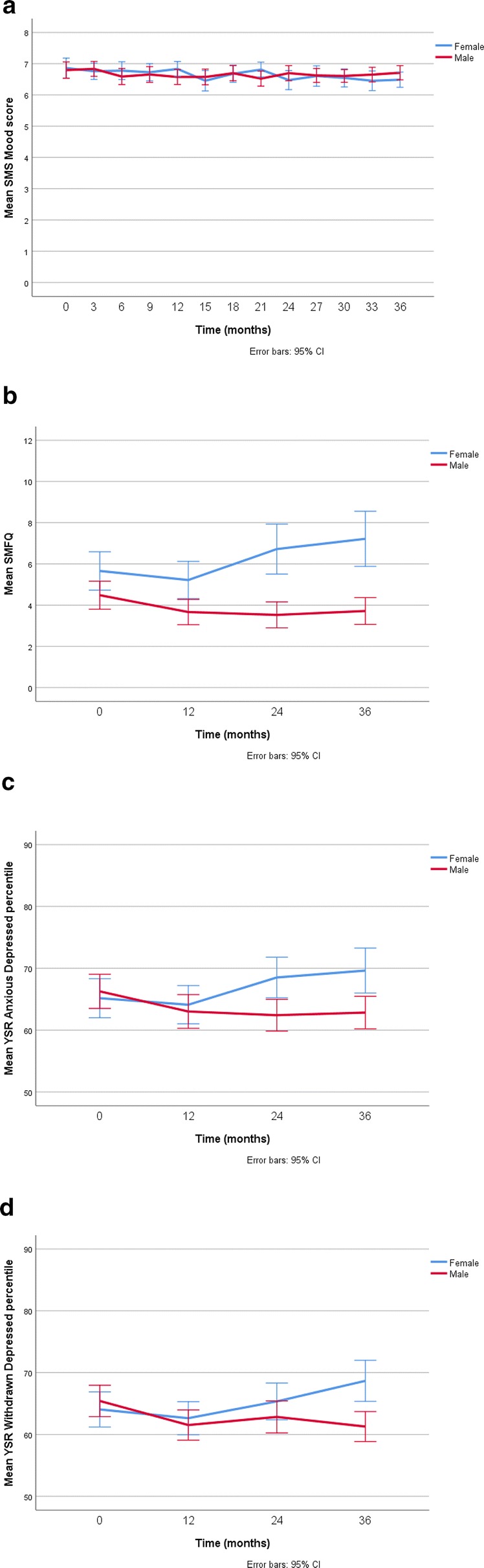
Mean mood rating scores over time measured by the four different scales (panels **a** to **d**), stratified by gender. *SMFQ* Short Mood and Feelings Questionnaire, *SMS* short message service, *YSR* youth self report

There was an 87% response rate to requests for SMS Mood ratings. Response rates varied as a percentage across the 13 collection points as follows; 86, 87, 93, 94, 94, 91, 88, 89, 91, 81, 83, 81, 78 (Cochran’s Q = 108, df = 12, p < 0.001). Response rates declined towards the end of the study period (logistic regression, where the dependent variable was SMS compliant (yes/no), and the predictor was time produced an odds ratio of 0.92 (95% CI 0.90–0.95).The mean SMS Mood score was 6.6 (SD 1.4, range 0–9). The mean SMS Mood score decreased over time for females but not males (see Table 2 and Fig. 1a). The percentage of SMS Mood scores falling in the ‘low’ range at each collection point did not vary significantly (range 5–9.3%, p = 0.50). One hundred and twenty-five participants (45%) had at least one SMS Mood score falling in the low range. Of these, 74 participants had a single low score and 51 had two or more low scores. Fifteen participants (5%) had clusters of low SMS Mood scores. Seven had such clusters commencing in year 1, three in year 2 and five in year 3.

**Table 2:** Trajectory of mood measures over 3 years of measurement

Mood measure	Sample	β	95% CI	p
SMS Mood	Females	− 0.03	− 0.05 to − 0.008	0.006
	Males	− 0.01	− 0.03 to 0.01	0.45
SMFQ	Females	− 0.16	0.05 to 0.27	0.006
	Males	− 0.06	− 0.13 to 0.01	0.07
YSR/AD	Females	0.45	0.11 to 0.79	0.010
	Males	− 0.27	− 0.52 to − 0.02	0.033
YSR/WD	Females	0.42	0.11 to 0.73	0.009
	Males	− 0.28	− 0.53 to − 0.02	0.032

SMS Mood n = 13 data points, other mood measures n = 4 data points

*SMS Mood* short messaging service mood measure, *SMFQ* short mood and feelings questionnaire, *YSR/AD* youth self report anxious depressed scale, *YSR/WD* youth self report withdrawn depressed scale

Mean SMFQ scores increased (more depression symptoms) over time for females but not males (see Table 2 and Fig. 1b). The percentage of participants scoring in the clinical range on the SMFQ at baseline and at subsequent 12 monthly data collection points was 12.6, 9.7, 14.4 and 17.3 respectively.

Mean YSR/AD scores increased (more depression symptoms) over time for females and decreased over time for males (see Table 2 and Fig. 1c). The percentage of participants scoring in the clinical range on the YSR/AD at baseline and at subsequent 12 monthly data collection points was 12.3, 11.9, 11.6 and 16.2 respectively.

Mean YSR/WD scores increased (more depression symptoms) over time for females and decreased over time for males (see Table 2 and Fig. 1d). The percentage of participants scoring in the clinical range on the YSR/WD at baseline and at subsequent 12 monthly data collection points was 6.9, 7.9, 8.3 and 11.2 respectively.

### Relationship between SMS Mood scores and other measures of mood

Linear regression analyses of continuous data found an inverse association between SMS Mood and SMFQ and YSR/WD, but not the YSR/AD (see Table 3). Subgroup analysis found an association between SMS Mood and the YSR/AD for females but not males. Risk analyses of binary data, expressed as odds ratios (OR), found an association between having a cluster of SMS Mood scores in the low range any time in the study period, and having an SMFQ score in the clinical range at both 24 (OR = 4.45, 95% CI 1.49 to 13.29) and 36 months (OR = 4.72, 95% CI 1.62 to 13.72). There was also a statistically significant association between having a cluster of SMS Mood scores in the low range any time in the study period and having a YSR/WD in the clinical range at 24 months (OR = 4.61, 95% CI 1.34 to 15.88).

**Table 3:** The association between SMS Mood score and SMFQ, YSR/AD and YSR/WD

	Model 1: dependent variable SMFQ			Model 2: dependent variable YSR/AD			Model 3: dependent variable YSR/WD		
	β	95% CI	p	β	95% CI	p	β	95% CI	p
Whole sample	− 0.35	− 0.63 to − 0.08	0.01	− 0.71	− 1.71 to.29	0.17	− 1.21	− 2.13 to − 0.29	0.01
Females	− 1.17	− 1.56 to − 0.77	< 0.001	− 2.28	− 3.51 to − 1.04	< 0.001	− 1.63	− 2.73 to − 0.52	0.004
Males	− 0.35	− 0.59 to − 0.12	0.003	− 0.72	− 1.70 to 0.26	0.15	− 1.21	− 2.13 to − 0.29	0.01

Parameter estimates are standardised beta coefficients, with 95% confidence intervals. Each model had SMS Mood score as a covariate, adjusted for gender and time. For the whole sample only, the model also included an interaction term (gender × SMS Mood score)

*SMS Mood* short messaging service mood measure, *SMFQ* Short Moods and Feelings Questionnaire, *YSR/AD* youth self report anxious depressed scale, *YSR/WD* youth self report withdrawn depressed scale

## Discussion

Participants who completed all four waves of the present study responded to 87% of the 3-monthly SMS text requests for a mood rating, demonstrating its feasibility for use in longitudinal research with young people. SMS Mood scores followed a similar trajectory over time as the SMFQ and YSR depression scales, with a worsening of mood among females while mood in males remained unchanged or even improved. The findings of the present study contrast with research using different depression scales which found gender difference remained constant over time [12, 13]), There was a robust and consistent relationship between SMS Mood scores and the depression scales in females, while the relationship was less consistent in males. The findings point to the SMS Mood scale being a more useful proxy measure for mood disturbance among adolescent females than males.

Having a cluster of SMS Mood scores in the low range occurred in only five percent of participants, but was associated with having SMFQ scores in the clinical range at 24 and 36 months. A series of low SMS Mood scores may be a precursor to depression identified by other means. If this is the case, a series of low SMS Mood scores could be used in longitudinal research to capture more accurately the onset of a depressive episode. The prevalence of clusters of low SMS Mood scores was similar to that of Major Depressive Disorder in adolescents, lending credibility to the proposition the two are associated. Isolated SMS Mood scores in the low range, on the other hand, were common (40% of participants), and their clinical significance is unknown. Single low SMS Mood scores may represent normal variation in mood, responses to environmental stress, biological events such as infection or possibly even hormonal change. In the present study, peri-menstrual dysphoria is unlikely to account for low SMS scores because females were no more likely than males to score in the low range on the SMS Mood scale (p = 0.42).

Strengths of the study include the high retention rate of participants over 3 years, and their high response rate to the request for SMS Mood ratings. Limitations to the study must also be acknowledged. The technology we used was, by current standards, primitive. Sophisticated apps to record and store mood information are now available for smartphones and other devices. The sample comprised volunteers from a specific region of rural Australia and may therefore not be representative of adolescents in general. The sample was modest in size for a community study, and therefore lacked the power to detect small effects. The study obtained only self-report of mood measures. It is known that adolescent reports of mood symptoms are inflated compared with corroborative reports from parents. However, this is a minor concern for the present study as we were interested in the relationship between measures and patterns over time rather than estimates of prevalence. The cut-score for low range SMS Mood scores was derived from ROC analyses of its association with the SMFQ, therefore introducing potential bias to subsequent analyses examining the relationship between the two measures when treated as binary variables. Owing to the low prevalence of clusters of low SMS Mood scores we were unable to perform more fine-grained analyses of their temporal association with the SMFQ and YSR.

## Conclusion

SMS Mood ratings represent a low cost means to augment but not replace assessment of mood obtained using standard instruments. Frequency of assessment can be tailored to the needs of the study. Clusters of low SMS Mood scores map on to high mood disturbance scores on standard mood measures, while the significance of isolated low SMS Mood scores is presently unknown.

## Data Availability

The datasets used and/or analysed during the current study are available from the corresponding author on reasonable request.

## Abbreviations

ARCHER: Adolescent Rural Cohort study of Hormones, health, Education, environments and Relationships
OR: odds ratio
PHQ-9: Patient Health Questionnaire-9
SMFQ: Short Mood and Feelings Questionnaire
SMS: short messaging service
YSR: youth self report
YSR/AD: youth self report anxious/depressed
YSR/WD: youth self report withdrawn/depressed
